# Metabolism-driven emerging acylation modifications in COPD: from elucidation of fundamental mechanisms to clinical diagnosis and treatment

**DOI:** 10.3389/fimmu.2026.1823993

**Published:** 2026-05-11

**Authors:** Guanglei Chen, Yunzhi Chen, Cancan Chu, Xing Zhu

**Affiliations:** School of Basic Medical Sciences of Traditional Chinese Medicine (Qihuang College), Guizhou University of Traditional Chinese Medicine, Guiyang, Guizhou, China

**Keywords:** chronic obstructive pulmonary disease, epigenetics, lactylation, lysine acylation, metabolic reprogramming, precision medicine

## Abstract

The progression of chronic obstructive pulmonary disease (COPD) is closely associated with metabolic reprogramming in pulmonary and immune cells. Under stresses such as cigarette smoke exposure, hypoxia, and infection, cells exhibit enhanced glycolysis, impaired mitochondrial oxidative metabolism, and altered tricarboxylic acid (TCA) cycle flux, resulting in abnormal accumulation of metabolites including lactate, succinate, and various acyl-coenzyme A species. These molecules, acting as acyl donors, drive emerging lysine acylation modifications (e.g., lactylation, succinylation, crotonylation), which play pivotal regulatory roles in airway inflammation, oxidative stress, and tissue remodeling by modulating chromatin states of histones or enzymatic activities of non-histone proteins. Studies have shown that histone lactylation (e.g., H3K14la, H4K12la) markedly induces senescence in pulmonary epithelial cells by activating p53 or CD38 expression and exacerbates pathological alterations, whereas succinylation and crotonylation show potential in regulating mitochondrial homeostasis and immune transcriptional programs. Non-histone acylation also plays an important role in feedback regulation of metabolic enzyme function and in proteostasis regulation. To achieve precision diagnosis and treatment, this review established an evidence-grading system based on strength of supporting evidence, indicating that high-strength sites such as lactylation should be prioritized for clinical translation. Future precision prevention and treatment of COPD should shift from mere description of modification abundance to causal validation of key sites, and should prioritize the development of smallmolecule drugs with isoform selectivity, in combination with pulmonary local delivery technologies to balance efficacy and safety. In addition, combined evaluation of specific metabolite levels and the acylation status of key proteins is expected to enable the development of biomarkers with greater predictive capacity, providing scientific support for molecular subtyping and precision intervention in COPD.

## 1 Introduction

Chronic obstructive pulmonary disease (COPD) is a pulmonary disease with marked heterogeneity, characterized by chronic respiratory symptoms caused by airway and/or alveolar abnormalities, leading to persistent, often progressive airflow limitation (1). Patients with COPD commonly present with exertional dyspnea, chronic cough, and sputum production; some patients may have concomitant wheezing, fatigue, and air trapping or dynamic hyperinflation, thereby markedly reducing quality of life (2). With the persistence of risk factors such as smoking exposure, occupational dust or chemical fumes, indoor and outdoor air pollution, together with an aging population, the burden of COPD continues to rise (3). The World Health Organization estimates that COPD causes substantial mortality annually, and the disease burden is more pronounced in low- and middle-income countries (4). COPD is characterized by a long disease course, high disability and mortality, and high treatment costs; in low- and middle-income countries, the economic and psychological burdens imposed on patients and their families continue to increase, making it an important public health problem that urgently needs to be addressed (5, 6). However, because its complex mechanisms of occurrence and progression have not been fully elucidated, current therapeutic approaches remain limited in their ability to improve prognosis and halt disease progression. Therefore, exploring new pathological mechanisms and precise intervention targets is of great significance for the prevention and treatment of COPD.

In recent years, a growing body of evidence has indicated that cellular metabolic reprogramming is closely associated with persistent airway inflammation and airway structural remodeling in COPD, and may play a key regulatory role in these processes (7–9). Under microenvironmental factors such as cigarette smoke extract (CSE), hypoxia, and pathogen infection, lung-resident cells such as alveolar epithelial cells, as well as immune cells including macrophages and neutrophils, can undergo remodeling of metabolic pathways, primarily manifested as enhanced glycolysis, impaired mitochondrial oxidative metabolism accompanied by alterations in tricarboxylic acid (TCA) cycle flux, and aberrant lipid metabolism (10, 11). These metabolic changes not only reshape cellular energy-supply patterns but also alter the availability of metabolites and their derived acyl-coenzyme A pools, such as lactate (12), succinate and its associated metabolic axes (13), acetyl-coenzyme A (14), and crotonyl-coenzyme A (15). Notably, beyond participating in energy metabolism to maintain cell survival, these molecules can also act as non-canonical signaling mediators and, as or after conversion into acyl donors, participate in protein post-translational modifications (PTMs), thereby directly regulating the structure and function of target proteins (16).

In this context, a class of metabolic state–regulated novel lysine acylation modifications has gradually been regarded as an important molecular link connecting metabolic reprogramming with COPD phenotypes (16–19). Unlike canonical lysine acetylation, modifications such as lysine lactylation, lysine succinylation, lysine crotonylation, and lysine 2-hydroxyisobutyrylation are highly sensitive to changes in the supply of lactate and the corresponding acyl-coenzyme A substrates, and can, to a certain extent, reflect remodeling of cellular metabolic fluxes (12, 17, 20– 23). Existing studies suggest (12, 18, 19, 24) that under COPD-related pathological stimuli, histone novel acylation modifications represented by lactylation can alter the expression of genes associated with inflammation and tissue remodeling by regulating chromatin states and transcriptional regulatory processes. Meanwhile, these modifications can also occur on non-histone proteins such as metabolic enzymes and affect their enzymatic activity or protein stability, thereby forming, under specific pathological contexts, a feedback regulatory loop between changes in metabolic state and alterations in cellular function (17, 23, 25, 26). Given the potential value of metabolism-driven novel lysine acylation modifications in precision diagnosis and treatment of COPD, this review will systematically summarize the metabolic sources and enzymatic basis of various modifications and their roles in pathological processes, aiming to provide a theoretical reference for clinical intervention and biomarker development based on the metabolic–modification regulatory axis by evaluating the strength of supporting evidence.

**Figure 1:**
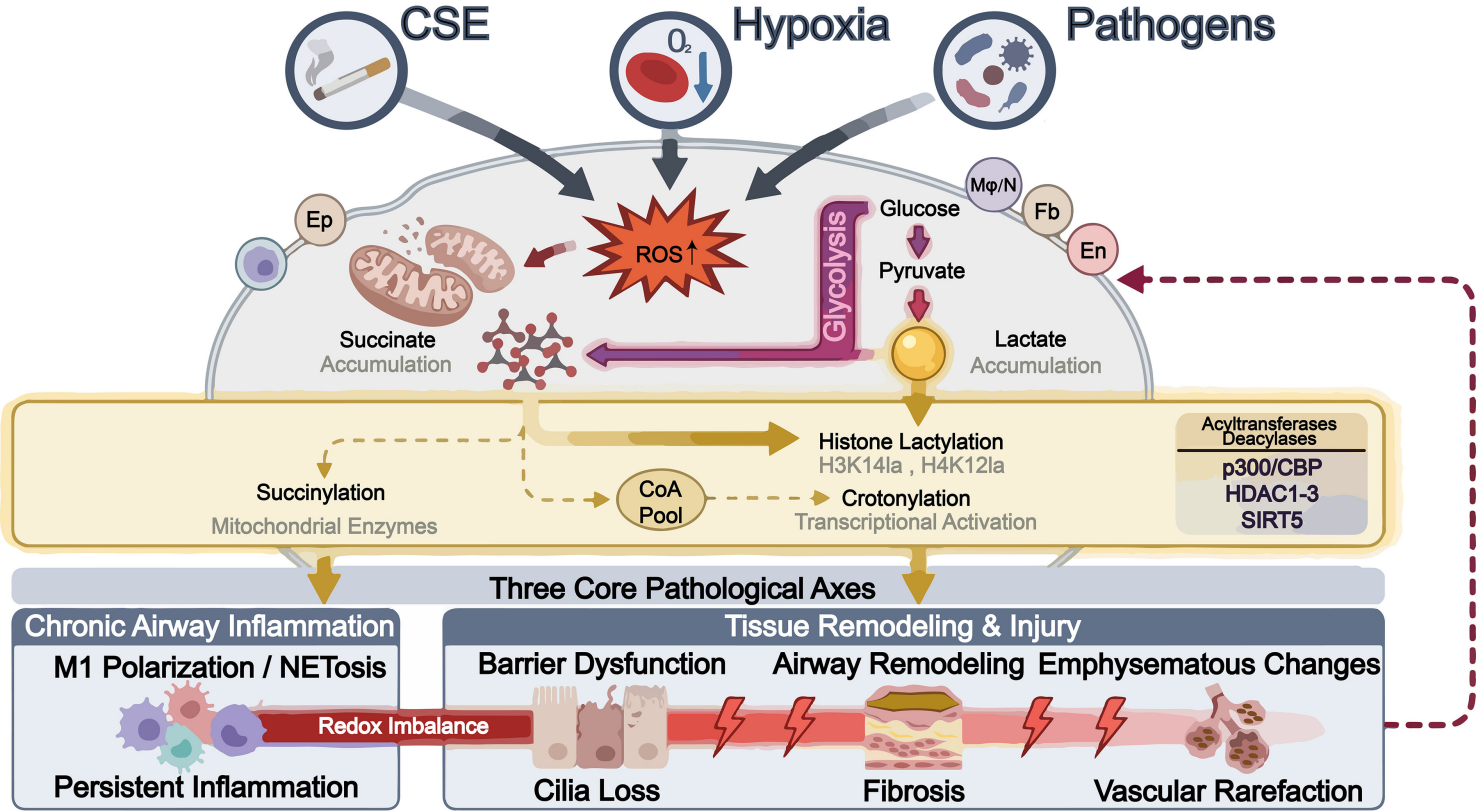
Schematic overview of the metabolic reprogramming–protein acylation axis and candidate feedback routes in COPD. External drivers such as cigarette smoke extract (CSE), hypoxia, and pathogen infection induce metabolic reprogramming in lung resident cells and immune cells, leading to ROS accumulation, enhanced glycolysis, mitochondrial dysfunction, and altered levels of lactate, succinate, and related acyl-coenzyme A pools. These metabolic changes promote emerging lysine acylation events under the regulation of p300/CBP, HDAC1–3, and SIRT5, including histone lactylation (H3K14la, H4K12la), succinylation of mitochondrial enzyme systems, and crotonylation associated with transcriptional activation. These acylation changes are linked to chronic airway inflammation, tissue remodeling and injury, and emphysematous/barrier-destructive changes in COPD. The dashed line indicates candidate feedback routes by which pathological outcomes may further intensify metabolic stress. Better-supported examples include lactylation-linked epithelial senescence and increased mitochondrial acylation burden under reduced SIRT5-related deacylation capacity, whereas similar feedback relationships for other acylation types remain provisional. Ep, epithelial cell; En, endothelial cell; Fb, fibroblast; Mφ/N, macrophage/neutrophil.

To visually summarize these complex interactions, we present a schematic framework in **Figure 1**, which illustrates how upstream metabolic drivers lead to specific protein acylations and subsequently fuel the three core pathological axes of COPD.

## 2 Cell type–specific features and inducing factors of metabolic reprogramming in COPD

Cellular metabolic reprogramming in COPD is not an isolated biochemical reaction, but rather the result of the combined effects of persistent exogenous stimuli and alterations in the local tissue microenvironment, and is closely associated with the chronic progression of the disease (7, 27, 28). Under normal pulmonary physiological conditions, cells maintain energy homeostasis and meet anabolic demands through metabolic networks; under pathological conditions of COPD, airway and lung parenchymal cells need to adjust their metabolic phenotype to adapt to long-term stress (7, 10, 27). This shift in metabolic patterns not only affects cell survival and functional maintenance but is also interrelated with key pathological processes such as enhanced inflammatory signaling, persistent oxidative stress, and tissue structural damage, thereby contributing to sustained disease progression (7, 10, 29–35).

CSE contains abundant free radicals and toxic components, which can damage the mitochondrial electron transport chain and increase reactive oxygen species (ROS) generation, thereby reducing the efficiency of the TCA cycle and oxidative phosphorylation (29–31, 36–38). With worsening airflow limitation and ventilation–perfusion mismatch, decreased local oxygen partial pressure can activate hypoxia-inducible factor 1 alpha (HIF-1α)-related signaling pathways, upregulate the expression of glycolysis-related genes, and impair oxidative metabolic capacity (32, 33, 39, 40). In addition, recurrent bacterial or viral infections can induce acute inflammatory responses and metabolic stress, further promoting ROS accumulation, making energy generation and redox homeostasis more prone to functional impairment (31, 34, 35, 41).

From the perspective of metabolic pathways, affected tissues and related cells in COPD often exhibit a state in which enhanced glycolytic activity coexists with impaired mitochondrial oxidative phosphorylation function, suggesting an altered mode of energy acquisition (7, 8, 10, 28, 36). This process may present as an aerobic glycolysis-like alteration, namely, even in the absence of complete oxygen deprivation, cells still tend to increase glycolytic flux to meet immediate energy and metabolic demands, accompanied by increased lactate production (7, 10, 32). Meanwhile, morphological and functional abnormalities such as mitochondrial fragmentation and decreased membrane potential are relatively common, which can lead to TCA flux remodeling and, at specific steps, constraints or local accumulation of intermediates (28, 30, 37). These changes can, to a certain extent, support the maintenance of basic functions under stress conditions, but may also cause fluctuations in metabolic intermediates, thereby affecting downstream signal transduction and cellular functional phenotypes (42).

In airway epithelial cells, metabolic reprogramming is associated with decreased barrier function and insufficient repair, which may increase the risk of persistent inflammation and promote structural alterations (37, 43–45). Airway epithelial cells typically rely on mitochondrial oxidative metabolism to support ciliary motility, maintenance of tight junctions, and regenerative repair after injury. When mitochondrial energy production declines and oxidative stress increases, ciliary function and epithelial integrity are more susceptible to damage. Metabolic abnormalities can also impair the proliferative and differentiation capacities of epithelial cells, reducing the efficiency of post-injury regeneration, and increase the production of pro-inflammatory factors and growth factors, thereby promoting processes related to airway remodeling (37, 43, 44).

Immune cells, especially macrophages and neutrophils, likewise exhibit significant metabolic changes in the COPD microenvironment, affecting the release of inflammatory mediators and effector functions (7, 8, 46). In lung tissues of patients with COPD, alveolar macrophages often exhibit suppressed mitochondrial respiration accompanied by enhanced glycolytic activity. This metabolic state is consistent with enhanced pro-inflammatory signaling and sustained release of inflammatory factors, manifested as elevated levels of interleukin 6 (IL-6) and tumor necrosis factor alpha (TNF-α); meanwhile, limited metabolic adaptability can coexist with decreased phagocytic clearance and tissue repair functions, making inflammation more difficult to effectively terminate (8, 36, 47). In neutrophils, altered metabolic states can be associated with prolonged survival time, degranulation, and enhanced oxidative burst, thereby aggravating protease-mediated lung tissue injury (46, 48, 49).

Changes in the metabolic phenotypes of structural cells such as pulmonary fibroblasts and vascular endothelial cells can influence the tissue remodeling process through increased extracellular matrix production and abnormalities in the vascular microenvironment (40, 44, 50–53). Previous studies have shown that remodeling of fibroblast metabolic pathways is associated with their differentiation into myofibroblasts and enhanced synthesis of extracellular matrix such as collagen, and may participate in the formation of small airway wall thickening and structural stiffening (27, 44, 53). On the other hand, constrained mitochondrial function and increased oxidative stress in pulmonary vascular endothelial cells can increase the propensity for apoptosis and weaken repair and angiogenic capacities; these changes are consistent with alveolar septal destruction and reduced capillary bed in emphysematous regions (40, 50–52). Metabolic abnormalities at the level of structural cells can promote the progression of COPD from a stage dominated by inflammation to a stage dominated by structural damage.

More importantly, global changes in metabolic flux can lead to concentration changes in metabolism-related molecules such as lactate, succinate, and acetyl-coenzyme A and crotonylcoenzyme A (7, 13, 17). Enhanced glycolysis can lead to lactate accumulation and is associated with increased levels of lysine lactylation; its acyl sources may involve pathways such as lactylcoenzyme A (lactyl-CoA) or lactoylglutathione (12, 54, 55). Meanwhile, constrained or remodeled TCA flux is often accompanied by changes in succinate levels and is associated with dynamic regulation of protein succinylation; changes in the availability of acetyl-coenzyme A and crotonylcoenzyme A can also influence the extent of multiple novel lysine acylation modifications (13– 15, 17, 25). Therefore, metabolic imbalance is not only reflected as abnormal energy metabolism but can also manifest as regulatory changes at the level of chemical modifications, further affecting processes of protein functional regulation, thereby providing a basis for subsequent discussion of metabolism-related acylation modification mechanisms. Because COPD-related metabolic and acylation changes are strongly cell-context dependent, observations derived from epithelial cells, macrophages, neutrophils, fibroblasts, endothelial cells, or peripheral blood cells should not be interpreted as interchangeable. Epithelial-cell findings are more directly related to barrier dysfunction, senescence, and abnormal repair; macrophage and neutrophil findings are more directly related to inflammatory persistence and impaired host defense; fibroblast and endothelialcell findings are more directly related to extracellular matrix remodeling and microvascular injury. Therefore, the disease relevance of a given acylation event should be interpreted together with its cellular source, rather than generalized across different COPD-relevant cell populations (8, 53).

## 3 Metabolism-driven acylation modifications

Metabolism-related acylation modifications refer to the phenomenon whereby alterations in cellular metabolic states, through factors such as acyl-donor supply, the activities of relevant enzymes, and the distribution of metabolites within subcellular compartments, affect the levels of protein posttranslational modifications, thereby linking changes in metabolic flux to changes in protein structure and function (16, 17). Compared with canonical signal transduction primarily characterized by kinase cascade reactions (17), lysine acylation in multiple contexts is more readily influenced by changes in the supply of metabolic substrates and cofactors, and thus can be used to characterize dynamic changes in cellular metabolic states.

The rates of generation and consumption of acyl-coenzyme A determine the substrate basis for lysine acylation reactions. The Michaelis constants of multiple lysine acyltransferases for acylcoenzyme A substrates fall within intracellular attainable concentration ranges, suggesting that even slight changes in the relative abundance of acyl-coenzyme A can affect enzymatic reaction rates and alter modification levels (56). In addition, under the relatively alkaline environment of the mitochondrial matrix and conditions in which certain acyl-coenzyme A species are highly reactive, modifications such as acetylation and succinylation can occur via non-enzymatic chemical reactions, the extent of which is related to local substrate concentrations and acid–base conditions (57). In the context of COPD-related metabolic reprogramming (20, 57, 58), such as enhanced glycolysis and reduced tricarboxylic acid cycle flux, the relative abundance and compartmental accessibility of specific acyl-coenzyme A species may change, thereby affecting the levels of different acylation modifications.

Beyond acyl-donor supply, levels of metabolic cofactors can also affect the formation and removal of acylation modifications. The activity of the sirtuin family of deacylases depends on nicotinamide adenine dinucleotide (NAD^+^), whereas the NAD^+^/NADH ratio can reflect cellular energy status and redox balance and influence the efficiency of deacylation reactions (59). The catalytic efficiency of the acetyltransferases p300 and CBP is influenced by acetyl-coenzyme A supply and enzyme conformational states (56). Under pathological conditions in which chronic inflammation and oxidative stress coexist, acylation modification levels can change accordingly with alterations in metabolic states.

Differences among subcellular compartments further affect the distribution of acylation modifications and their functional consequences. Because charged molecules such as acetylcoenzyme A cannot freely cross biological membranes, their generation and utilization within different organelles depend on compartmentalized metabolic processes. For example, acetylcoenzyme A required for nuclear acetylation can be generated after citrate transport via ATP-citrate lyase, and under specific conditions can also be supplied by the pyruvate dehydrogenase complex localized in the nucleus (14). In contrast, the acylation levels of mitochondrial proteins are more readily influenced by acyl-coenzyme A levels within the mitochondrial matrix (57). Therefore, in analyzing COPD-related acylation modifications, differences in metabolite distribution among the nucleus, cytosol, and mitochondria should be considered simultaneously.

Mechanistic studies of metabolism-related acylation require, in addition to correlational descriptions, the introduction of intervention strategies to support causal inference. Although panacylation antibodies can be used for preliminary screening, their specificity and lot-to-lot variability may affect result consistency, and confirmation using orthogonal methods is advisable (60). Highresolution liquid chromatography–tandem mass spectrometry remains the primary technical approach for modification site identification; when combined with stable isotope labeling and dataindependent acquisition strategies, site-level quantification and estimation of modification stoichiometry can be achieved, thereby enhancing biological interpretability (61). On this basis, genetic modulation of metabolic enzymes or relevant enzymes, or supplementation with exogenous metabolites followed by assessment of site-specific modifications and functional phenotypic changes, can further clarify the causal relationship between metabolic alterations and acylation modifications. Overall, current studies on novel acylation modifications mainly focus on functional validation, specific detection, and quantitative analysis. To more clearly compare the characteristics and scope of application of each technical strategy and their performance in COPD research, they are summarized in **Table 1**. Leveraging these detection and validation approaches, researchers can identify functional modification sites in complex lung tissues of COPD that are induced by metabolic alterations.

**Table 1:** Technical approaches and evaluation of research on novel acylation modifications.

Evaluation dimension	Site-specific antibody-based detection	Mass spectrometry–based proteomics identification	Mutant-based mimetics	Pharmacological/genetic intervention
Method category	Immunodetection: site-specific antibodies (62, 63)	Proteomics: modified-peptide enrichment + high-resolution LC-MS/MS (64)	Molecular biology: site-directed mutagenesis, expression, and functional assays (65)	Functional intervention: small-molecule inhibitors/CRISPR/knockout/knockdown (66, 67)
Questions addressed	Do known sites differ? What is the spatial distribution within tissues? (62, 63)	Which sites are acylated? How do site abundances change? (12, 17, 64)	Is a given site necessary for function? (65)	Do upstream (metabolism–enzyme–modification) changes lead to downstream phenotypes? (66, 67)
Resolution	Epitope-centered at the site; IHC provides cell-level information (62, 63)	Site-level achievable; covers multiple proteins/multiple sites (12, 64)	Single-site level (65)	Mostly enzyme-/pathway-level interventions; site-level confirmation requires combination with MS (66, 67)
Quantification	Mostly semi-quantitative/relative (62, 63)	Both relative and absolute: SILAC (68), TMT (69), PRM (70), AQUA (71)	Primarily functional phenotypes; does not directly provide true modification stoichiometry (65)	Primarily intervention–phenotype; site quantification depends on PRM/AQUA or site-antibody back-validation (70, 71)
Key quality control	Antibody specificity and batch consistency; KO, peptide competition, and orthogonal validation recommended (62, 63)	FDR control, AScore (70, 72), isotope calibration (68, 71), PRM (70, 72)	Consistency of mutant expression/localization; prevent analogical distortion due to structural differences (65)	Inhibitor selectivity, genetic rescue, and parallel multi-omics analyses (66)
Sample requirements	High compatibility with FFPE; fresh/frozen tissues and PBMC also applicable (62, 63)	Prefer fresh/frozen; FFPE is more sensitive for recovery; starting material depends on enrichment strategy (64)	Mainly cell lines/primary cells; not directly applicable to patient tissues (65)	Mainly cell and animal models; aligned with COPD-relevant models (73–75)
Throughput and timeline	High throughput, low cost, short cycle; IHC involves slide-reading time (62, 63)	High throughput, high platform cost, medium-to-long cycle; can reduce cost with PRM (70, 76)	Moderate throughput, moderate cycle (65)	Moderate throughput, long cycle; but most critical for the causal chain (66, 67)
Strength of causal inference	Primarily associative	Strong associative evidence, not equivalent to causality	Strong site-level mechanism; requires closed-loop validation (65)	Closest to causality when combined rescue experiments are consistent (66, 67)
Feasibility in COPD	Highly compatible with clinical diagnostic workflows and FFPE samples (73–75, 77)	Feasible for research; targeted PRM has translational potential (70, 76)	Mechanism-oriented; needs linkage to COPDrelevant phenotypes	Consistent with known abnormalities in COPD deacetylation axes (73–75)
Translational potential	Site-specific IHC as tissue biomarkers; can be integrated with prognosis (62, 63)	Can be developed into clinical targeted quantitative assays (12, 70, 76)	Translation mainly as mechanistic support (65)	Oriented toward drug targets (66, 67, 73–75)
Main limitations	Strong antibody dependence; cannot cover unknown sites (62, 63)	Low stoichiometry; challenges in isomer discrimination; complex analysis (64, 72)	Mimetic ≠ authentic: differences in stereochemistry/charge/volume; KQ may be overestimated (65)	Insufficient selectivity; broad transcriptional effects; off-target side effects (66, 67)
Recommended combinations	Post-discovery clinical validation: MS/literature → site-specific antibody IHC/WB → cohort validation (12, 62, 63)	Integrated discovery–validation: unbiased MS → PRM targeted quantification → antibody-based spatial backvalidation (64, 68–71, 76)	Mechanistic strengthening: site-directed mutagenesis + PRM-based authentic site detection + functional phenotype assessment (65)	Causal closed loop: upstream enzyme intervention → site monitoring → phenotype assessment (66, 67, 73–75)

In summary, metabolism-related lysine acylation modifications are jointly influenced by acyl-donor supply, the activities of relevant enzymes, and subcellular compartmental distribution. Thus, in the context of metabolic reprogramming in COPD, changes in the supply of different metabolic intermediates and acyl-coenzyme A species may selectively affect the levels and site distributions of specific acylation modifications, and further associate with cellular phenotypic changes. Based on these mechanistic points, the subsequent content will focus on novel lysine acylation types reported in COPD, summarizing their major metabolic sources, potential regulatory steps, and corresponding relationships with disease-associated phenotypes.

## 4 Novel types of lysine acylation in COPD

In the context of COPD-related metabolic reprogramming, changes in the intracellular availability of lactate and multiple acyl-coenzyme A species, together with changes in the activity of the relevant catalytic enzymes and deacylases, can alter the levels of multiple lysine acylations other than acetylation, thereby affecting chromatin-associated transcription and the function of metabolic enzymes. Compared with lysine acetylation, these acylations differ in charge, side-chain structure, and reader recognition, and may therefore differ in their effects on chromatin accessibility, transcriptional output, and protein activity (17). Partial overlap in metabolic inputs and regulatory enzymes indicates a biochemical relationship between classical lysine acetylation and emerging acylation pathways, but does not indicate functional equivalence (17, 78). This section summarizes the acylation types that have been observed in samples from patients with COPD and in smoke exposure-related models (see **Table 2**), and outlines their potential metabolic sources and functional associations.

**Table 2:** Characteristics of metabolism-driven novel lysine acylation modifications in COPD pathology.

Acylation type	Metabolic driver & subcellular location	Experimental models & disease context	Detection strategy	Modified proteins/Sites	Associated regulatory pathways	Phenotypic associations	References
Lactylation (Kla)	Drivers: lactate accumulation due to increased glycolytic flux; altered levels of lactylcoenzyme A (lactyl-CoA) or lactoylglutathione. Localization: primarily focused on nuclear histones; non-histone modifications also exist.	1. 1-nitropyrene (1-NP) model: simulates COPD-like pathology; concurrent increases in glycolysis and histone lactylation observed. 2. Alveolar type II epithelial cells (AT2): cellular senescence model. 3. Cigarette smoke exposure: chronic mouse model and peripheral blood mononuclear cells (PBMC) from patients with COPD. 4. Environmental exposure: benzo[a]pyrene (BaP) activates the aryl hydrocarbon receptor (AhR) axis.	1. Immunological detection: site-specific antibodies (H3K14la, H3K18la) combined with immunoblotting/immunofl uorescence. 2. Chromatin analysis: CUT&Tag, ChIP-qPCR, and ChIPseq.	1. H3K14la: significantly upregulated after 1-NP exposure. 2. H4K12la: a key modification site during AT2 cell senescence. 3. H3K18la: increased levels in PBMC from patients with COPD.	1. p53 transcriptional activation: H3K14la associated with upregulated p53 transcription. 2. CD38–NAD^+^ axis: H4K12la upregulates CD38 expression, consuming NAD^+^; p300/CBP inhibitor (A-485) can block this process. 3. Inflammatory gene regulation: butyrate treatment associated with decreased H3K18la and attenuation of related inflammatory gene sets.	1. Cellular senescence: epithelial cell cycle arrest and senescence-associated secretory phenotype (SASP). 2. Tissue injury: worsened lung histopathology scores. 3. Inflammation regulation: butyrate intervention improves lung function and reduces inflammatory cell infiltration.	(12, 18, 19, 27, 79, 80)
Succinylation (Ksucc)	Driver: supply of succinylcoenzyme A (succinyl- CoA), a tricarboxylic acid (TCA) cycle intermediate. Localization: highly enriched in mitochondrial proteins. De-modifying enzyme: SIRT5 has desuccinylase activity.	1. Normal lung tissue: baseline modified-proteome datasets established. 2. Disease models: epithelial cells treated with cigarette smoke extract (CSE) and COPD clinical samples. Note: current studies mainly focus on SIRT5 expression levels and enzymatic activity changes; direct functional validation of Ksucc sites is relatively limited.	1. Omics identification: anti-succinyl-lysine antibody enrichment combined with LCMS/MS. 2. Functional studies: coimmunoprecipitation, subcellular fractionation, and assays of apoptosisrelated indices. 3. Clinical samples: qRT-PCR to measure SIRT5 transcript levels; immunoblotting did not assess SIRT5 protein.	1. Lung-tissue modification mapping: numerous mitochondrial protein modification sites identified in normal tissues. 2. Diseaserelated: key COPD-specific Ksucc driver sites have not yet been established; existing findings largely involve SIRT5-mediated FOXO3 deacetylation (not succinylation) regulation.	1. Mitochondrial homeostasis: involved in regulating mitochondrial metabolic enzyme activity. 2. SIRT5–FOXO3 axis: although a deacetylation mechanism, it reflects the core role of SIRT5 in oxidative stress and apoptosis.	1. Apoptosis: CSEinduced programmed cell death. 2. Oxidative stress: mitochondrial dysfunction and reactive oxygen species (ROS) accumulation. 3. Decline in lung function: SIRT5 transcript levels in severe COPD are lower than in controls, with significant differences between mild and severe groups.	(81, 82, 85–88)
Crotonylation (Kcr)	Driver: availability of crotonyl-coenzyme A (crotonyl-CoA); fatty acid metabolism and lysine degradation. Localization: histones and non-histone proteins in transcriptionally active regions. Enzymatic basis: p300 has crotonyltransferase activity.	1. Clinical samples: PBMC from patients with COPD complicated by type II respiratory failure. 2. Exposure factor: crotonaldehyde in cigarette smoke may serve as a precursor (to be confirmed).	1. Proteomics: TMT-based quantitative labeling + crotonylation enrichment + high-resolution LCMS/MS. 2. Bioinformatics: MaxQuant quantitative analysis combined with GO/KEGG functional annotation.	1. Differential modification analysis: 32 upregulated sites (involving 23 proteins) and 914 downregulated sites (involving 295 proteins) reported. 2. Protein overlap: some Kcr-modified proteins also show altered protein expression levels.	1. Immune-related pathway enrichment in crotonylome/proteome analysis: differentially modified proteins significantly enriched in IL-17 signaling and antigen processing and presentation–related processes. 2. Association with transcriptional activity: histone Kcr is generally positively correlated with transcriptionally active states.	1. Systemic immune association observed in PBMC-based profiling; causal relevance to lung pathology remains unvalidated. 2. Immune dysfunction: functional annotation indicates enrichment in antigen processing and presentation and immunerelated processes.	(15, 89, 90, 92, 101, 102)
2-Hydroxyisobutyryl ation (Khib)	Driver: nutritional status determines levels of 2hydroxyisobutyrylcoenzyme A (2-Hib-CoA). Localization: histones and glycolytic enzyme systems. Enzymatic basis: p300 mediates the modification reaction.	Current status: in the COPD field, discussion is mainly at the review and hypothesis stage, lacking systematic site identification and functional intervention studies in lung tissue or specific cell types.	1. Standard strategy: antiKhib antibody enrichment combined with LCMS/MS. 2. Enzymatic intervention: genetic or pharmacological inhibition of p300 combined with metabolic phenotyping analysis.	Basic research reference: in non-COPD experimental systems, lysine 2hydroxyisobutyrylation has been detected in glycolysisrelated enzymes such as ENO1, and its modification level is regulated by p300 status.	Functional inference from nonCOPD models; COPD-specific sitefunction validation is unavailable.	Metabolic reprogramming: potentially participates in the shift of cellular energy metabolic patterns, but its specific pathological contribution in COPD remains to be confirmed.	(21, 22, 94, 95)
Other acylations (Kmal, Kglu, Kbhb)	Drivers: levels of malonylcoenzyme A (malonylCoA), glutaryl-coenzyme A (glutaryl-CoA), and β-hydroxybutyrate (BHB). De-modifying enzyme: SIRT5 has multiple deacylation activities (malonyl/succinyl/glutaryl).	1. Fasting/ketosis models: increased BHB induces Kbhb. 2. COPD status: limited numbers of site-level studies in COPD lung tissue and classical models; currently mostly mechanistic inferences under the background of metabolic dysregulation.	1. Omics and validation: enrichment-based MS detection and immunoblotting with site-specific antibodies. 2. Metabolic intervention: fasting treatment or exogenous BHB supplementation combined with HDAC activity assays.	1. Histone Kbhb: increases with rising BHB concentration, with site specificity. 2. Glutarylated proteins: negatively regulated by SIRT5.	1. Starvation-response transcription: Kbhb marks promoter regions of specific genes. 2. Epigenetic crosstalk: BHB can inhibit class I HDACs, leading to non-specific increases in histone acetylation.	1. Stress tolerance: enhances cellular adaptation to oxidative stress or nutrient deprivation. 2. Mechanistic inference from non-COPD metabolic models; COPDspecific site-level and functional evidence remains limited.	(82, 96–99)

### 4.1 Lysine lactylation (Kla)

In a 1-nitropyrene-induced COPD model, enhanced glycolysis was accompanied by increased histone lactylation levels (19). Studies have shown that increased H3K14la promotes transcription of the p53 gene and induces epithelial cell senescence. Inhibition of lactate production reduces H3K14la levels and alleviates the senescence phenotype. Another study reported H4K12la as a major modification site in alveolar type II epithelial cells (79); this modification regulates CD38 expression, leading to NAD^+^ consumption and promoting cellular senescence. Use of p300/CBP inhibitors reduces H4K12la and ameliorates COPD-related phenotypes; within the same metabolic axis, CD38 inhibition and NAD^+^ precursor supplementation can alleviate NAD^+^ depletion and improve senescence-related indices, but their direct effects on H4K12la still require site-level validation. A case–control study (18) suggested that benzo[a]pyrene-related exposure is associated with AhR activation and correlates with increased H4K12la in lung tissue and elevated senescencerelated indices. In immune cells (80), increased H3K18la or global lactylation levels have been observed in chronic cigarette smoke exposure models and in peripheral blood mononuclear cells (PBMC) from patients with COPD. Experimental results showed that butyrate treatment reduced global lysine lactylation levels and H3K18la levels, accompanied by attenuated transcriptional signatures of inflammation-related signaling gene sets and inhibition of NFκB activation; however, the association between changes in NFκB activation and alterations in H3K18la still requires further verification.

### 4.2 Lysine succinylation (Ksucc)

Lysine succinylation uses succinyl-CoA as the donor and can markedly alter the electrostatic property of the lysine side chain, thereby affecting protein interactions and conformation (81). Given that succinyl-CoA mainly derives from mitochondrial metabolic processes, Ksucc is relatively abundant on mitochondrial proteins; the desuccinylase SIRT5 participates in maintaining and regulating its levels (82). In COPD, mitochondrial dysfunction may alter succinyl-CoA supply and desuccinylation-related activity, thereby changing overall Ksucc levels and site distributions. Systematic identification of succinylation in normal human lung tissue has been performed, and omics-level changes in lung succinylation have also been reported in inhalational lung injury models (83–85); in contrast, systematic studies of site-level identification, regulatory factors, and functional effects of Ksucc in classical COPD animal models and patient lung tissue remain relatively limited. Reports regarding SIRT5 changes under COPD conditions are inconsistent: in pulmonary epithelial cells, cigarette smoke extract treatment upregulated SIRT5 and reduced acetylation of FOXO3 at the K271 and K290 sites; this change was accompanied by enhanced nuclear localization of FOXO3 and reduced apoptosis, suggesting that SIRT5-mediated deacetylation processes are associated with stress-defense–related phenotypes in epithelial cells (86). In addition, clinical samples and cellular experiments have also shown decreased SIRT5 expression under COPD conditions (87, 88). These findings suggest that the current COPD-related evidence remains insufficient to support a uniform direction of SIRT5 alteration. SIRT5 is more appropriately defined as a context-dependent regulatory factor, and its relationship with protein desuccinylation, deacetylation, and downstream phenotypes may vary depending on factors such as cell type, exposure conditions, and disease stage.

### 4.3 Lysine crotonylation (Kcr)

Lysine crotonylation (Kcr) uses crotonyl-coenzyme A (crotonyl-CoA) as the acyl donor. Given that crotonyl-CoA supply is influenced by cellular metabolic states, Kcr may participate in linking metabolic changes to transcriptional regulation. In multiple cell types, histone Kcr is often enriched at transcriptionally active regulatory elements and is correlated with increased gene expression (89). p300 has been confirmed to possess enzymatic activity catalyzing the formation of histone crotonylation, providing an enzymatic basis for the generation of this modification (15). In COPDrelated studies, proteomic analyses showed differential distribution of Kcr modification sites in PBMC from patients with COPD complicated by type II respiratory failure, suggesting that Kcr may be associated with systemic immune status (90). Regarding upstream metabolic factors, crotonaldehyde in cigarette smoke may affect crotonyl-CoA supply (15, 91, 92); however, direct evidence is still lacking to support that it increases intracellular crotonyl-CoA levels in lung cells and further enhances protein or histone Kcr. Given the sensitivity of Kcr to crotonyl-CoA levels and the fact that intranuclear sources of crotonyl-CoA have not been fully clarified, these inferences still require further experimental validation. At present, COPD-related evidence for Kcr is derived mainly from observational studies (90). Existing reports on COPD with type II respiratory failure are based primarily on differential crotonylome and proteomic analyses of PBMCs, while sitespecific functional evidence is still lacking, and no causal association with pulmonary pathological changes has been established. Therefore, the pathological significance of Kcr in COPD remains to be further clarified.

### 4.4 Lysine 2-hydroxyisobutyrylation (Khib)

Lysine 2-hydroxyisobutyrylation is a widely distributed modification, and the generation of its donor, 2-hydroxyisobutyryl-coenzyme A, is related to nutritional status (21). p300 has enzymatic activity that promotes this modification, and Khib has been identified on multiple glycolytic enzymes, suggesting a potential effect on metabolic reaction rates (93, 94). However, these findings are derived mainly from non-COPD systems, and COPD-specific site-level identification, perturbation-rescue data, and phenotype-linked validation remain lacking. At present, systematic identification and functional studies of Khib in lung tissue from patients with COPD or in animal models remain limited (95). Further studies are needed to define its specific sites and biological roles in lung tissue and key cell types such as epithelial cells and macrophages.

### 4.5 Other lysine acylation types

Modifications such as lysine malonylation, lysine glutarylation, and lysine β-hydroxybutyrylation are also influenced by cellular metabolic states (82, 96–98). Previous studies showed that elevated levels of the ketone body β-hydroxybutyrate can promote increased histone lysine βhydroxybutyrylation (Kbhb) and are correlated with upregulated expression of genes associated with starvation responses (98); in addition, β-hydroxybutyrate can also upregulate the expression of oxidative stress–related genes such as FOXO3A and MT2 by inhibiting class I histone deacetylases (HDACs) (99). To date, in COPD lung tissue and classical COPD animal models, systematic proteomic identification and site-function studies of novel acylation modifications such as lysine malonylation (Kmal), lysine glutarylation (Kglu), and lysine β-hydroxybutyrylation (Kbhb) remain relatively limited, and for some modification types, reliable direct evidence is still lacking. Accordingly, the current discussion of Kmal, Kglu, and Kbhb in COPD should be regarded as biochemical plausibility rather than disease-specific causal evidence.

### 4.6 Interplay between classical lysine acetylation and emerging lysine acylations in COPD

Although emerging lysine acylation modifications are considered to participate in COPD-related metabolic remodeling, their relationship with canonical lysine acetylation remains unclear. Previous studies (20, 81) have identified extensive overlap between lysine succinylation and lysine acetylation across multiple proteins and lysine sites, particularly in metabolic enzymes, and multiple acyl marks may coexist within the same protein system. However, in COPD, it remains unknown whether the same lysine residue undergoes competitive occupancy, sequential switching, or functional synergy, as site-resolved occupancy data for shared sites and direct functional validation are still lacking. At the level of regulatory enzymes (15, 94, 100), p300/CBP, in addition to mediating acetylation, also participates in histone crotonylation, lysine 2-hydroxyisobutyrylation, and certain histone succinylation events; HDAC1–3 and members of the sirtuin family can remove distinct acyl marks under different substrate and subcellular compartment conditions (78, 82). These findings suggest crosstalk between canonical acetylation pathways and emerging acylation pathways, but there is still no evidence supporting functional equivalence among different acyl marks. In COPD research, downregulation of SIRT1 and increased acetylation of RelA/p65 remain among the more directly supported inflammation-related mechanisms (75). In contrast, evidence related to emerging acylation modifications is derived mainly from observations at histone sites, changes in overall abundance, or studies in non-COPD systems. SIRT5 has also not shown consistent conclusions in COPD: first, in epithelial cells exposed to cigarette smoke extract, SIRT5 is associated with FOXO3 deacetylation and reduced apoptosis (86); second, low SIRT5 expression has also been associated with mitochondrial dysfunction and more severe disease phenotypes (88). Therefore, SIRT5 is more likely to act as a context-dependent regulatory factor, with its effects influenced by factors such as cell type, exposure duration, disease stage, mitochondrial status, and level of analysis, while direct evidence for its coordinated regulation of acetylation and succinylation at COPD-relevant sites is still lacking.

## 5 Histone lysine acylation and chromatin-associated transcriptional regulation

Histone lysine acylation can alter the physicochemical properties of the nucleosome surface and its protein–protein interaction interfaces, thereby regulating transcriptional activity in promoter- and enhancer-proximal regions, and is correlated with cellular transcriptional states (17). Among the reported novel acylations, lactylation and crotonylation predominantly occur on histone lysine residues and mainly affect nucleosome properties by weakening the positive charge of lysine; succinylation, in addition to reducing positive charge, can introduce a net negative charge and increase side-chain volume, and is therefore generally considered to exert a more pronounced impact on nucleosome structure and stability (12, 17, 89, 100). Notably, novel acylation sites can be distributed both on the N-terminal tails of histones and on key residues within the globular domains. For example, H3K122succ is located at the nucleosome–DNA contact interface, and its succinylation can reduce nucleosome stability and enhance *in vitro* transcription reactions (100, 103).

Based on studies such as chromatin immunoprecipitation sequencing, lactylation marks including H3K18la can be enriched near genes with higher transcriptional activity in multiple cell types and are positively correlated with the expression levels of the corresponding genes (12, 104). Crotonylation can also be enriched in promoter-proximal regions and is associated with an active transcriptional state. From a mechanistic perspective, p300 possesses histone crotonyltransferase activity, and p300-catalyzed histone crotonylation can promote transcription reactions in *in vitro* systems (15). Therefore, under pathological conditions in which chronic inflammation and metabolic alterations coexist, novel histone acylations may participate in regulating the expression of specific gene sets; the scope of target genes and cell-type dependence still need to be defined within specific experimental systems.

The transcriptional effects of novel histone acylations are closely associated with reader-domain proteins. The representative reader domain for crotonylation is the YEATS domain; YEATSdomain proteins can bind crotonylated histones and form functional coupling with transcription elongation–related complexes, thereby promoting an active transcriptional state (101, 105). Bromodomain recognition of acetylation is relatively canonical; some bromodomains can also bind specific non-acetylated acylated peptides, but this is still insufficient to explain a general recognition mechanism for novel acylations (106). In transcription elongation regulation, the interaction between BRD4 and PTEFb can enhance phosphorylation of the RNA polymerase II carboxy-terminal domain and promote transcription elongation, providing clear biochemical evidence for the involvement of acetylation-associated reader proteins in transcription elongation regulation. Regarding lactylation, studies have reported that DPF2 can act as an effector protein of H3K14la, and results also suggest a functional association between BRD4 and site-specific lactylation; however, its generality and disease-related specificity still require further validation across different models (107–109). This difference from classical bromodomain-centered acetylation recognition suggests that emerging histone acylations may not simply reproduce acetylation-dependent transcriptional effects.

In addition to reader proteins, the substrate selectivity of catalytic enzymes and the intracellular availability of substrates jointly influence the formation strength and spatial distribution of different types and sites of histone acylation. p300/CBP can participate in the formation of H3K122succ, and succinylation at this site is associated with promoter activity; regarding removal, desuccinylation of H3K122succ has been reported in studies related to SIRT7 and SIRT5, suggesting that multienzyme coordinated regulation may exist under different cellular conditions (100, 103). Another study showed that KAT2A can enter the nucleus in coordination with a mitochondriaassociated complex and catalyze H3K79succ, thereby affecting gene expression in regions proximal to transcription start sites (110). These results indicate that histone succinylation is not solely derived from passive chemical reactions, and that its formation and removal have a clear enzymatic regulatory basis.

In COPD-related studies, histone lactylation has been associated with epithelial cell senescence and disease phenotypes in certain models, corresponding to site-level changes in transcriptional regulation, suggesting that under conditions of chronic inflammation and metabolic alterations in COPD, novel histone acylations may participate in maintaining specific transcriptional states (16, 79). However, a substantial proportion of conclusions in existing studies are primarily based on correlational analyses between global modification levels and transcriptomic changes. The sufficiency and necessity of site-specific modifications in function still require more rigorous functional studies for validation. To further delineate how different types of histone acylation modifications coordinately participate in transcriptional remodeling during COPD pathology, **Table 3** summarizes the association features of these novel modification marks with specific transcriptional programs, genomic contexts, and related co-regulatory factors.

**Table 3:** Associations between novel histone lysine acylation and transcriptional regulatory programs in COPD.

Transcriptional program	Key histone acylation mark	Genomic/Chromatin context	Reader domain protein/Co-regulator	Representative targets (Genes/Gene sets)	Disease relevance	References
Basal program associated with high transcriptional activity	H3K18la	1. Active promoters and regions proximal to transcription start sites; 2. Can also mark tissue-specific active enhancers and is positively correlated with transcriptional activity	1. p300/CBP; 2. HDAC1–3	1. Housekeeping gene sets; 2. Gene sets associated with tissuespecific enhancers	1. Provides histone-level evidence linking metabolic state to chromatin transcriptional activity; 2. In COPD, constitutes a transferable mechanistic basis and requires further investigation at the levels of lung tissue and cell types	(12, 78, 104)
COPD inflammation-related immune transcriptional program	H3K18la	1. Changes in lactylation enrichment at promoters/proximal regions of inflammation-related pathway genes; 2. Coexistence of increased enrichment at the SOCS2 locus and decreased enrichment at loci related to multiple inflammatory pathways	1. Mechanistic emphasis mainly on changes in metabolite levels and histone lactylation levels; 2. Delactylases (HDAC1–3) as potential regulatory nodes	1. Gene sets in pathways such as MAPK, JAKSTAT, and Th17 differentiation; 2. SOCS2	Provides evidence in COPD mouse models and patient PBMC showing increased lactylation and its association with inflammation alleviation	(78, 80)
Program associated with active transcription	H3K18cr, H3K9cr	1. Enriched in promoter-proximal regions and at transcription start sites; 2. Associated with an active transcriptional state	1. YEATS domain proteins (AF9, YEATS2, etc.) as representative reader modules; 2. p300 has histone crotonyltransferase activity and can promote *in vitro* transcription reactions	1. Gene sets with active transcription; 2. Gene expression modules related to transcription elongation/activation	Overall, direct site-level and causalchain evidence in COPD is insufficient, but it provides an epigenetic basis for transcriptional maintenance mechanisms under chronic inflammation and metabolic alterations	(15, 101, 111)
Regulation of nucleosome stability and transcriptional output	H3K122succ	1. Histone dimer–DNA contact interface within the nucleosome; 2. Its succinylation can reduce nucleosome stability and enhance *in vitro* transcription reactions	1. p300/CBP (associated with H3K122succ formation); 2. SIRT5 and SIRT7	1. Output of *in vitro* transcription systems; 2. Sites associated with active gene promoters	1. In COPD research, supports that succinylation exerts a more pronounced impact on nucleosome physicochemical properties; 2. Evidence in specific COPD tissues and cell types remains limited	(100, 103)
Coordinated regulatory program of mitochondrial metabolism and nuclear transcription initiation	H3K79succ	1. Enriched in regions proximal to transcription start sites; 2. Associated with nuclear import of the intranuclear α-KGDH complex and the catalytic activity of KAT2A	1. KAT2A (succinyltransferase activity associated with site formation); 2. αKGDH complex (associated with nuclear localization and substrate supply)	KAT2A-dependent target gene sets	In the context of COPD-related metabolic remodeling, this suggests the involvement of nuclear metabolic enzyme complexes in histone acylation regulation	(110)
COPD/model-related lung epithelial senescence transcriptional program	H3K14la	1. Enhanced lactylation at the p53 promoter region, associated with p53 transcriptional upregulation and senescence phenotypes	1. In this COPD model study, emphasis was placed on lactate accumulation and upregulation of lactylation; 2. DPF2 has been validated as an effector protein of H3K14la in other systems and can serve as a candidate co-regulator	1. TP53; 2. Cellular senescence–related gene sets (e.g., p21/CDKN1A)	Histone lactylation is associated with COPD-like phenotypes (premature senescence/pulmonary function and structural alterations)	(19, 109)
COPD alveolar type II epithelial cell senescence and NAD metabolism–related program	H4K12la	1. Epigenetic regulatory changes associated with the CD38–NAD^+^ signaling axis	1. p300 inhibitor A485 as an intervention node; 2. CD38 inhibitor 78c and NMN supplementation used for functional-chain validation	1. CD38; 2. NAD^+^ metabolism–related gene sets; 3. Cellular senescence–related phenotypic indices	Association among lactylation sites, metabolic pathways, and cellular senescence phenotypes in COPD	(12, 79)

Systematic integration of the above transcriptional programs indicates that novel histone lysine acylations can affect nucleosome stability by altering the charge properties and side-chain volume of lysine, and regulate transcriptional activity in promoter- and enhancer-proximal regions. Their transcriptional effects are closely related to the specificity of modification sites, the binding features of reader-domain proteins, and the substrate selectivity of catalytic enzymes, and are simultaneously constrained by the intracellular availability of metabolic substrates. Under pathological conditions such as COPD, in which chronic inflammation and metabolic abnormalities coexist, modifications such as lactylation are correlated with epithelial cell senescence phenotypes and transcriptional changes. However, existing studies are mostly based on correspondence between global modification levels and transcriptomic alterations, and rigorous causal validation of the sufficiency and necessity of site-specific modifications remains lacking. Future studies should combine site-specific regulatory strategies with *in vivo* tissue-specific validation to clarify the functional boundaries of different acylation types in COPD-related transcriptional regulation, and to improve the credibility and translational potential of epigenetic regulatory intervention target selection.

## 6 Non-histone lysine acylation: regulation of protein function and metabolic feedback

Under COPD-related metabolic stress, histone acylation and non-histone acylation may represent two mechanistically distinct but metabolically coupled response layers. Upstream changes such as increased lactate availability, redistribution of acyl-coenzyme A pools, and mitochondrial dysfunction may simultaneously affect the acylation status of chromatin-associated and nonchromatin proteins (17). At the histone level, these metabolic alterations are linked to transcriptional programs related to cellular senescence, inflammation, and NAD^+^ metabolism, as illustrated by H3K14la-associated upregulation of p53 transcription and H4K12la-associated activation of the CD38–NAD^+^ axis in COPD-related epithelial models (19, 79). At the non-histone level, the same metabolic context may increase the acylation burden of glycolytic enzymes, mitochondrial metabolic proteins, and respiratory-chain–related systems, thereby affecting metabolic flux, electron transfer efficiency, ROS handling, and inflammatory signaling output (25, 54, 112). A more cautious interpretation is that histone acylation is linked mainly to stabilization of disease-relevant transcriptional states, whereas non-histone acylation is associated mainly with metabolic and redox execution processes that may further influence metabolite availability (17, 25, 54). Thus, the two levels should not be viewed as completely independent, although in current COPD studies their relationship is still supported mainly at the level of pathway convergence rather than direct site-specific causal transfer. At present, the strongest evidence remains concentrated on lactylation-linked epithelial senescence and SIRT5-related mitochondrial stress responses.

Under pathological conditions, non-histone lysine acylation may serve as a relatively rapid mode of protein regulation, allowing COPD-related metabolic changes to be reflected more directly as alterations in protein functional states and thereby participating in metabolic feedback regulation (96, 113).

Mechanistically, unlike histone modifications that primarily affect chromatin structure, non-histone lysine acylation mainly regulates enzymatic activity and molecular interactions by altering local physicochemical properties of proteins. Under physiological conditions, the amino group of the lysine side chain is usually positively charged, which helps maintain local salt bridges and substrate positioning (96). Acetylation and lactylation can neutralize the positive charge, alter the local electrostatic environment, and affect conformational stability of proteins (12, 54). By contrast, succinylation, malonylation, and glutarylation, in addition to neutralizing positive charge, may introduce negative charge and increase side-chain volume, thereby more markedly affecting the spatial geometric features of active sites and the assembly stability of complexes (20, 96). Therefore, these modifications at the site level can alter enzymatic catalytic efficiency, substrate specificity, and the strength of protein complex interactions.

On this basis, lysine acylation of metabolic enzymes can provide a more direct protein-level intervention point for regulating metabolic activity. Proteomic studies have shown that key enzymes in glycolysis and the tricarboxylic acid cycle harbor widespread succinylation modifications, and the corresponding sites show a high degree of overlap with acetylation sites (20). However, in COPD-related systems, such overlap should currently be interpreted as evidence of possible crosstalk rather than direct proof of competitive occupancy or coordinated site-specific regulation. Meanwhile, the alkaline environment of the mitochondrial matrix and relatively high levels of acyl-coenzyme A can promote non-enzymatic modifications without enzyme involvement, suggesting that metabolite abundance itself may influence modification levels (57). Under pathological conditions, if acylation at specific sites reduces the activity of rate-limiting enzymes, upstream intermediate metabolites may further accumulate and maintain high substrate levels, thereby making metabolic pattern shifts easier to sustain; such relatively stable changes may serve as an explanatory direction for the maintenance of metabolic abnormalities in COPD, but the scope of applicability still needs to be delimited in conjunction with site-function validation (96).

Under conditions of persistent metabolic abnormalities, the association between increased mitochondrial protein acylation burden and altered respiratory chain function is of greater interest. Loss of SIRT5 or impaired SIRT5 activity can lead to increased succinylation and glutarylation levels of mitochondrial proteins (including proteins related to electron transport chain complexes) (25, 97). Accumulation of these modifications may affect complex assembly stability and electron transfer efficiency, accompanied by decreased membrane potential and increased generation of ROS (57). In COPD-derived cellular and animal models, mitochondrial dysfunction and elevated ROS have been reported in multiple studies, thereby providing a disease context for the association among metabolic alterations, changes in protein acylation, and oxidative stress (114, 115).

Against this background of mitochondrial functional alterations and oxidative stress, the magnitude and persistence of inflammatory signaling may also be influenced by lysine acylation states. In the context of smoking exposure and COPD, decreased SIRT1 levels are often accompanied by increased acetylation levels of NFκB subunits (e.g., p65) (75, 116). Under these conditions, acylation modifications of signaling proteins can enhance inflammatory signal output by affecting protein stability, nuclear translocation efficiency, or the assembly state of transcriptional complexes. Meanwhile, lactate accumulation induced by hypoxia and inflammatory stimuli can promote nonhistone lactylation (12), thereby creating a closer correspondence between changes in metabolic states and enhanced inflammatory signaling; however, this correspondence still needs to define its necessity and sufficiency at the level of specific sites. At present, this SIRT1–RelA/p65 acetylation axis remains more directly supported in COPD than any corresponding non-acetyl acylation mechanism involving NFκB signaling.

In addition to metabolism- and inflammation-related proteins, processes related to proteostasis may also be influenced by lysine acylation. Lysine is a major acceptor site for ubiquitination, and modifications such as acetylation can competitively inhibit ubiquitination mediated by ligases such as Mdm2 at specific sites, thereby prolonging the half-life of key proteins such as p53 (117). Meanwhile, the NAD^+^-dependent deacetylase SIRT1 participates in autophagic flux regulation and affects maintenance of cellular homeostasis under nutrient restriction conditions (118). Therefore, through influencing acylation and deacylation levels, metabolic states may alter competitive relationships between acylation and ubiquitination and exert persistent regulatory effects at the levels of protein degradation and autophagy, thereby affecting cellular survival under stress conditions.

Given the broad distribution of non-histone acylation sites and their marked functional heterogeneity, their biological significance needs to be delineated through rigorous validation strategies (representative target proteins and effects are shown in **Table 4**). Lysine-to-arginine (K to R) mutations are often used to mimic a de-modified state retaining a positive charge, whereas lysine-to-glutamine (K to Q) mutations are often used to mimic a charge-neutralized state; however, such substitution mutations cannot fully recapitulate the stereochemical features of authentic modifications and may lead to biased effect estimation (65). For modifications that introduce negative charge and increase side-chain volume, simple substitution with acidic amino acids likewise cannot reproduce their spatial effects. Therefore, site-specific mutagenesis studies should be conducted in combination with *in vitro* enzymatic kinetic measurements, quantitative detection of modification levels, and cellular functional assays (96). In addition, defining the subcellular compartment in which the modification occurs is likewise important for elucidating its correspondence with specific metabolic events (57).

**Table 4:** Representative non-histone lysine acylation target proteins, modification types, and functional effects under COPD-related metabolic states.

Target protein	Function	Acylation type	Effect on activity	Mechanism	References
ALDOA	Key glycolytic enzyme	Lysine lactylation (K147)	Enzymatic activity ↓, glycolytic flux ↓	K147 is located in a region proximal to substrate-binding– related structures; site-specific lactylation inhibits ALDOA catalytic function and leads to accumulation of upstream intermediates	(119)
Glycolytic enzyme group	Maintenance of glycolytic metabolic output	Lysine lactylation (LactoylLys, non-enzymatic formation)	Glycolytic output ↓	Lactoylglutathione mediates non-enzymatic transfer of the lactyl group to lysine side chains; GLO2 deficiency leads to increased lactoylglutathione and markedly elevated LactoylLys, accompanied by decreased major glycolytic metabolites	(54)
Mitochondrial protein group	Protein set related to oxidative metabolism and energy production	Lysine acetylation; lysine succinylation (nonenzymatic formation)	Acylation level ↑	The alkaline environment of the mitochondrial matrix and relatively high levels of reactive acyl-coenzyme A drive acetylation and succinylation, increasing in a time- and dose- dependent manner	(57)
3-hydroxy-3-methylglutarylcoenzyme A synthase 2 (HMGCS2)	Rate-limiting enzyme for ketogenesis	Lysine succinylation	Enzymatic activity ↓	A hypersuccinylated state associated with SIRT5 deficiency inhibits HMGCS2; acidic substitution mutations at key hypersuccinylation sites can markedly inhibit enzymatic activity	(25)
SIRT5	Mitochondrial deacylase	Substrate lysine succinylation; substrate lysine glutarylation	Substrate acylation level ↓	As a desuccinylase and deglutarylase, SIRT5 maintains mitochondrial protein acylation burden at relatively low levels and regulates the modification status of multiple classes of metabolic proteins	(25, 97)
RelA p65 (NFκB subunit)	Inflammation-related transcriptional regulation	Lysine acetylation (including the K310 site)	Transcriptional activation function ↑	p300 or CBP mediates RelA acetylation; K310 acetylation is necessary for full transcriptional activation; SIRT1 can deacetylate K310 and suppress NFκB-dependent gene expression; in COPD-related samples, decreased SIRT1 levels are accompanied by increased RelA acetylation	(75, 120–122)
p53	Stress response– and growth inhibition–related transcription factor	Lysine acetylation	Ubiquitination ↓, protein stability ↑	C-terminal acetylation of p53 can directly inhibit Mdm2- mediated ubiquitination, prolonging the half-life of p53	(117)
ATG5, ATG7, ATG8 (LC3)	Key autophagy molecules	Lysine acetylation	Autophagic flux ↑ after deacetylation	Sirt1 deacetylates ATG proteins and promotes starvationinduced autophagy; defective deacetylation function is associated with insufficient autophagy activation	(118)
Ku proteins (Ku70-related system)	Components of DNA repair–related protein complexes	Substitution mutagenesis mimicking acetylation sites (K to Q, K to R)	Effect estimation is inflated for K to Q	Computational simulations show that K-to-Q mutation reduces DNA-binding free energy, whereas binding energy in a fully acetylated state is close to that of the wild type, indicating that K-to-Q substitution is not stereochemically equivalent to authentic acetylation	(65)

## 7 Unified categorization of research findings and determination of translational prioritization

Throughout this review, disease relevance is discussed at three distinct levels: site-resolved functional evidence in COPD models, associative evidence in COPD samples or models, and mechanistic inference from non-COPD systems. These levels should not be interpreted as equivalent.

Previous studies on lysine acylation modifications in COPD have mostly focused on single molecules, specific sites, or limited cellular models. Owing to differences in experimental materials, exposure modalities, disease stages, and detection methods, conclusions across studies are often difficult to compare on the same scale, which constrains our ability to synthesize shared disease patterns. To improve comparability, we categorized acylation changes in histones and non-histone proteins according to key pathological processes during disease progression. Restating existing findings using unified terminology and adjudication criteria can minimize the interference of expression-related discrepancies with integrative assessment.

During COPD progression, chronic airway inflammation, redox imbalance, and tissue structural remodeling typically occur in parallel and are closely associated with symptom exacerbation and disease-course evolution. Accordingly, assigning reported acylation changes to the pathological processes above facilitates establishing a consistent descriptive framework between molecular mechanisms and clinical phenotypes (see **Table 5**). Meanwhile, alterations in metabolic status often span multiple pathological processes and participate in regulation partly through covalent modifications such as lysine acylation. Therefore, examining the three categories of pathological processes together is more conducive to explaining convergent phenotypic trends across different studies.

**Table 5:** Systematic associations and grading of emerging lysine acylation events across the three core pathological processes of COPD.

Key acylation events and molecular nodes	Research context and temporal characteristics	Association with chronic airway inflammation	Association with redox imbalance	Association with tissue remodeling	Cross-study consistency and validation status	Evidence level	References
Kla; increased H3K14la	Chronic exposure animal model; lung epithelial cells; with intervention controls	Co-occurs with inflammatory cell infiltration and senescence-related phenotypes	Not directly addressed	Not directly addressed	The relationship between the site and transcriptional regulation is relatively clear; clinical stratification data are insufficient	II	(19)
Kla; increased H4K12la	COPD model; alveolar type II epithelial cells; with pharmacological intervention	Predominantly senescence-related phenotypes; inflammatory components are mostly indirectly associated	Associated with NAD^+^ changes	Indirect association	Site screening and intervention validation are relatively sufficient; clinical and longitudinal data remain limited	II	(79)
Kla; AhR-related increased H4K12la	Cross-sectional human lung tissue evidence with exposure-context cell studies; longitudinal stage-specific evidence remains lacking	Descriptions related to inflammatory factors are reported in the study conclusions	Not directly addressed	Indirect association	Correlations in patient tissues are relatively clear; longitudinal data are insufficient	III	(18)
Kla; increased H3K18la	Chronic smoking exposure model and patient PBMC analyses; evidence is immune-cell dominant and should not be directly generalized to lung structural cells	Associated with altered immuneinflammatory pathways and cellular infiltration	Not directly addressed	Not directly addressed	Both animal and patient peripheral blood data are available; site function still requires further refinement	II	(80)
Ksucc; global-level changes	COPD-specific data are limited; more commonly observed in other lung injury settings or basic research	Indirect association	Predominantly inferred from mitochondrial metabolism-related evidence	Indirect association	Data on sites and disease stratification are insufficient	IV	(81–85)
Ksucc-related regulation: altered SIRT5	Primarily acute epithelial CSE models, with additional patient expression data; the relationship between acute stress responses and chronic diseasemaintaining signatures remains unresolved	Indirect association	Associated with cytoprotective phenomena related to oxidative stress	Not directly addressed	Limited number of COPD studies; conclusions are substantially influenced by model conditions	III	(86–88)
Kcr; peripheral blood proteomics differences Primarily single-cohort observational evidence in PBMCs; no site-specific perturbation or orthogonal functional validation in COPD lung models.	COPD with type II respiratory failure; PBMC; small sample size	Mainly described in relation to systemic immunity	Not directly addressed	Not directly addressed	Primarily observational; mechanistic data are insufficient	III	(90)
Khib; modification of glycolysisrelated proteins Primarily mechanistic evidence derived from non-COPD systems; no COPD-specific site-level replication or intervention-rescue data.	General basic research across multiple diseases	Not directly addressed	Not directly addressed	Not directly addressed	Biochemical and chromatin-level data are relatively sufficient; disease specificity is insufficient	IV	(15, 89, 92)
Khib; modification of glycolysis- related proteins	Mainly nutritional and metabolic models	Not directly addressed	Not directly addressed	Indirect association	*In vivo* replication and sitelevel data in COPD are insufficient	IV	(21, 94, 95)
Others (Kmal, Kbhb, etc.) Primarily biochemical plausibility and non-COPD evidence; no disease-specific validation in COPD tissues or models.	Mainly basic metabolism and other disease models	Not directly addressed	Indirect association	Not directly addressed	Insufficient COPD-specific replication	IV	(96–99)

Taking lactylation studies as an example, changes at several sites have shown good reproducibility under different experimental conditions and are highly consistent with phenomena such as epithelial cell senescence and enhanced inflammation. For instance, in a chronic environmental exposure model, upregulation of H3K14la is associated with enhanced p53 transcription and lung epithelial cell senescence (19); a study in alveolar type II epithelial cells found that increased H4K12la can promote cellular senescence and aggravate COPD-like changes by affecting CD38- and NAD^+^related signaling (79); another exposure study suggested that AhR activation is associated with increased H4K12la, and in patient lung tissue it shows a correlation with cellular senescence indicators (18). In addition, although global lactylation and H3K18la levels decreased under intervention in a smoking exposure model, increased H3K18la in peripheral blood mononuclear cells from patients has been reported (80). These results suggest that certain lactylation sites may simultaneously participate in inflammatory responses, oxidative stress injury, and tissue structural changes, but their specific roles remain constrained by cell type, exposure context, and disease stage. Notably, there is substantial inter-individual heterogeneity among COPD patients. The magnitude of change of the same modification may differ dramatically across sample sources, exposure histories, or disease stages. Therefore, when synthesizing conclusions, the applicable scope of these findings must be explicitly indicated, and results from specific experimental models must not be mechanically generalized to all patients. On this basis, when evaluating which research targets have greater translational value, priority should be given to indicators that recur across different experimental conditions and show consistent associations with two or more pathological processes. Compared with isolated statistical differences observed in a single experiment, such cross-condition consistency of association is more informative for guiding subsequent allocation of research resources.

In addition to differences in sample and spatial origin, temporal factors also limit the applicability of conclusions. Transient modification changes induced by acute stimuli are usually closer to immediate metabolic responses, whereas stable changes formed under chronic exposure more closely reflect the molecular state during the disease maintenance phase. Therefore, when proposing mechanistic hypotheses and intervention concepts, early changes and late-stage concomitant injury should be strictly distinguished in longitudinal observations or stage-specific models, to avoid interpretive bias caused by mismatched temporal scales. Accordingly, findings from acute smoke, hypoxia, or infection models are discussed in this review mainly as stress-response signals, whereas findings from chronic exposure models and patient-derived samples are interpreted more cautiously as candidate disease-maintaining signatures. Therapeutic implications are considered only within this temporal context.

Under the above premises, we applied a graded strength-of-evidence framework to standardize the ranking of candidate sites and molecules (see **Table 6**). The grading criteria reflect a stepwise relationship, with primary adjudication bases including whether the cellular source is clearly defined, whether site identification is sufficient, whether *in vivo* results are reproducible, and whether findings are consistent with patient stratification indices. When basic research findings are stably correlated with patients’ clinical indices, their translational potential is relatively higher; by contrast, findings that remain at the stage of observations in a single model are more suitable as starting points for subsequent studies and should not serve as the primary basis for mechanistic conclusions and clinical concepts.

**Table 6:** Criteria for grading strength of research support and translational prioritization.

Grade	Positioning and translational orientation	Key validation criteria	Major limitations
I	Strong support (prioritized for translational research)	Cell source is clearly defined and reproducible in COPD-relevant cell types; modification sites are confirmed by mass spectrometry or orthogonal methods; animal-model findings show consistent correlations with patient stratification indices; functional validation is available in at least two key pathological processes	Confounding factors still need to be assessed; large-cohort and long-term follow-up data may be insufficient
II	Moderate support (prioritized for mechanistic research)	Modulation of the modification can reverse specific cellular phenotypes; target molecules or sites are relatively clear but lack comprehensive *in vivo* and clinical stratification correspondence; a relatively clear relationship between the modification and phenotype has been established within a single key pathological process	Insufficient clinical extension data; lack of longitudinal observations; model-specific bias may be present
III	Weak support (candidate direction)	Mainly based on cross-sectional studies or correlative observations in a single model; relies on non–site-specific antibodies or indirect indicators; lacks replication in independent cohorts or by orthogonal methods	Regulatory direction is unclear; highly constrained by conditions; relatively high risk of bias
IV	Background support (theoretical inference)	Extrapolated from non-COPD models or biochemical principles; lacks definition of specific sites, cell types, or disease context	Only indicates plausibility and is difficult to use directly for disease interpretation and translational prioritization

## 8 Translational and therapeutic implications: from selection of intervention targets to biomarker applications

In the field of COPD research, whether lysine acylation modifications can advance to clinical practice hinges on whether they can explain disease heterogeneity and guide therapeutic decisionmaking. At the basic research stage, attention is typically focused on screening differential sites; however, upon entering the translational stage, the research emphasis must shift to the robustness of regulatory molecules. Intervention targets that can enter validation procedures should exhibit consistent patterns of change across clinical samples from different sources and across different pathological stages. Only when the modification status of a specific molecule can establish clear quantitative associations with core phenotypes such as inflammatory infiltration, oxidative injury, and tissue remodeling, and can effectively predict the risk of acute exacerbations or disease progression, can its clinical application value be truly established.

Intervention strategies targeting such molecules need to balance efficacy and safety. Although regulating systemic metabolism can alter substrate concentrations and thereby correct multiple classes of acylation modifications, given that COPD patients are often accompanied by systemic metabolic dysregulation and smoking-induced stress responses, such generalized regulatory strategies are highly prone to generating response bias across different tissues and increasing the risk of off-target adverse effects. Therefore, at the current stage, metabolic interventions should mainly be used to validate mechanisms and identify populations that may benefit. To develop such approaches into clinical therapeutic regimens, drug delivery routes must be improved not only to reduce systemic exposure, but also to address uneven pulmonary deposition, local clearance, and restricted access to diseased microenvironments, and on this basis to define a clear safe dose range for specific patient subgroups.

Compared with macroscopic metabolic regulation, direct intervention of modification enzyme systems or recognition proteins is more targeted. However, COPD pathological processes involve multiple major cell types, including airway epithelial cells, macrophages, and fibroblasts, and the same change in enzymatic activity may produce markedly different, or even opposite, biological effects in different cellular contexts. Accordingly, targets identified in acute epithelial or immune stress models should not be assumed to remain suitable intervention points during established chronic disease, and therapeutic windows should be defined in a stage-specific and cell-typespecific manner. Enzymes represented by p300/CBP or the SIRT family have very broad substrate spectra; if a drug lacks sufficient isoform selectivity, it can readily trigger widespread transcriptional dysregulation. Moreover, isoform selectivity does not by itself ensure biological selectivity within the COPD lung microenvironment. Even when catalytic selectivity is improved, enzymes such as p300/CBP and sirtuins remain embedded in broad transcriptional and metabolic regulatory networks, and the net effect of inhibiting the same target may differ across epithelial cells, macrophages, fibroblasts, and endothelial cells. In addition, pulmonary local delivery may reduce systemic exposure, but does not guarantee uniform deposition, cell-selective uptake, or sustained drug exposure in diseased lung regions, because airway obstruction, mucus retention, mucociliary clearance, and phagocytic removal can all affect local drug distribution. Therefore, drug development should focus on improving enzymatic selectivity and dose–response relationships, and use patient-derived primary cells to evaluate the biological net benefit after longterm administration across multiple COPD-relevant cell types. Only when multiple pathological indices are consistently improved across different cellular models does an intervention strategy have a scientific basis to advance toward clinical trials.

Beyond serving as therapeutic targets, lysine acylation signatures also hold substantial promise for biomarker development. In view of current limitations such as insufficient sensitivity of clinical early-warning indicators and the singularity of assessment approaches, combined evaluation of specific metabolite levels and the acylation status of key proteins would more objectively reflect the dynamic evolution of the disease. In this context, the development focus should shift from mechanistic elucidation to the stability of detection technologies. Relevant indicators must undergo rigorous clinical analytical validation to ensure high reproducibility in multicenter validation cohorts. In addition, whether the assay is noninvasive (e.g., blood or sputum samples) and the correspondence between assay results and clinical outcomes are key determinants of whether these biomarkers can be truly implemented.

In summary, the transition from basic research to clinical application should be grounded in reverse analysis of clinical needs. Translational research is not a mechanical transfer of laboratory conclusions, but should follow a rigorous process of reproducibility substantiation, phenotypeassociation analyses, and upfront safety assessment. Only by ensuring that detection protocols are standardized and fully validated can findings related to lysine acylation modifications be translated into stable, reproducible diagnostic and therapeutic tools, providing objective evidence for optimizing clinical interventions for COPD.

## 9 Summary and perspectives

Alterations in protein post-translational modifications induced by metabolic reprogramming provide important clues for investigating the initiation and progression of COPD. Existing studies indicate that accumulated metabolites such as lactate, succinate, and specific acyl–coenzyme A species are not only consequences of disordered energy metabolism, but also act as signaling molecules involved in the regulation of gene transcription and modulation of protein activity. These epigenetic modifications triggered by microenvironmental metabolic stress show significant associations with the chronic persistence of airway inflammation, premature senescence of epithelial cells, and structural damage to lung tissue. Although emerging modifications such as lactylation and succinylation have demonstrated preliminary biological effects in experimental models, more rigorous validation at multiple levels is still required to translate them into clinical diagnosis and treatment.

Future research should shift from merely describing modification abundance to providing causal explanations for key sites. Although high-resolution mass spectrometry has identified a large number of differentially modified sites, because protein functions differ markedly across cell types and subcellular localizations, site accumulation alone cannot directly indicate their contribution to pathological processes. Therefore, subsequent work should focus on using site-specific mutagenesis or precise enzymatic interventions to clarify the direct effects of specific acylation modifications on pro-inflammatory signal transduction or the efficiency of the mitochondrial respiratory chain. Only when the biological significance of specific sites is reproducibly validated across multiple independent experimental systems will the relevant molecules have scientific value as targets for pharmacological intervention.

This evolution from discovery of basic principles to determination of clinical value requires investigators to directly confront the high heterogeneity of COPD. Given the substantial differences among patients in specimen sources, smoking exposure contexts, and disease stages, the implications of the same modification signature may not be consistent across different subgroups. Future efforts should be directed toward establishing a standardized evaluation scheme and, under a unified terminology and adjudication framework, grading the strength of evidence for different acylation events. By integrating large-scale clinical data with longitudinal observations of disease course, it will be possible to identify indicators that are highly robust and tightly associated with clinical phenotypes, thereby providing objective evidence for molecular subtyping of the disease. With respect to the development of clinical intervention modalities, it is necessary to balance therapeutic benefit against off-target risk. This balance cannot be inferred from enzyme selectivity alone, because selective inhibition of broadly acting regulators may still yield divergent biological effects across different COPD-relevant cell populations and uneven exposure across diseased lung regions. Although modulation of global metabolism can correct modification levels, because COPD patients often have systemic impairment of metabolic homeostasis, generalized regulatory approaches can readily lead to adverse effects in extrapulmonary tissues. Therefore, developing small-molecule antagonists with isoform selectivity, in combination with pulmonary local delivery technologies to increase local effective concentrations, represents a feasible direction for achieving precision intervention. In addition, combined evaluation of specific metabolite levels and the acylation status of key proteins is expected to enable the development of biomarkers with greater predictive capacity than traditional indicators, thereby improving monitoring of acute exacerbation risk.

In summary, the transition from mechanistic elucidation to clinical application must follow rigorous procedures for reproducibility substantiation and phenotype-association evaluation. As detection protocols become standardized and external validation systems are improved, findings related to lysine acylation modifications will gradually be translated into stable, reproducible diagnostic and therapeutic tools. Continued exploration of metabolic and modification-regulatory processes is expected to provide scientific support for optimizing COPD treatment regimens, delaying respiratory functional failure, and improving patient prognosis.

## Glossary

1-NP: 1-nitropyrene
2-Hib-CoA: 2-hydroxyisobutyryl-coenzyme A
AhR: aryl hydrocarbon receptor
ALDOA: aldolase, fructose-bisphosphate A
AQUA: absolute quantification peptide
AT2: alveolar type II epithelial cells
ATG: autophagy-related
BaP: benzo[a]pyrene
BHB: β-hydroxybutyrate
BRD4: bromodomain-containing protein 4
CBP: CREB-binding protein
CD38: cluster of differentiation 38
COPD: chronic obstructive pulmonary disease
crotonyl-CoA: crotonyl-coenzyme A
CSE: cigarette smoke extract
FFPE: formalin-fixed, paraffin-embedded
GLO2: glyoxalase II
glutaryl-CoA: glutaryl-coenzyme A
H3K14la: histone H3 lysine 14 lactylation
H3K18cr: histone H3 lysine 18 crotonylation
H3K18la: histone H3 lysine 18 lactylation
H3K9cr: histone H3 lysine 9 crotonylation
H4K12la: histone H4 lysine 12 lactylation
HDAC: histone deacetylase
HIF-1α: hypoxia-inducible factor 1-alpha
HMGCS2: 3-hydroxy-3-methylglutaryl-coenzyme A synthase 2
KAT2A: lysine acetyltransferase 2A
Kbhb: lysine β-hydroxybutyrylation
Kcr: lysine crotonylation
Kglu: lysine glutarylation
Khib: lysine 2-hydroxyisobutyrylation
Kla: lysine lactylation
Kmal: lysine malonylation
KQ: lysine-to-glutamine mutant
Ksucc: lysine succinylation
lactyl-CoA: lactyl-coenzyme A
LC3: microtubule-associated proteins 1A/1B light chain 3
malonyl-CoA: malonyl-coenzyme A
MS: mass spectrometry
MT2: metallothionein 2
NAD⁺: nicotinamide adenine dinucleotide
NADH: nicotinamide adenine dinucleotide (reduced form)
NMN: nicotinamide mononucleotide;
p21/CDKN1A: cyclin-dependent kinase inhibitor 1A
PBMC: peripheral blood mononuclear cells
PRM: parallel reaction monitoring
PTEFb: positive transcription elongation factor b
PTMs: post-translational modifications
ROS: reactive oxygen species
SASP: senescence-associated secretory phenotype
SILAC: stable isotope labeling by amino acids in cell culture
SIRT: sirtuin
SOCS2: suppressor of cytokine signaling 2
succinyl-CoA: succinyl-coenzyme A
TCA: tricarboxylic acid cycle
TMT: tandem mass tag
YEATS: YEATS domain
α-KGDH: α-ketoglutarate dehydrogenase
